# Ocular Siderosis Secondary to Retained Intraocular Foreign Body: A Case Report

**DOI:** 10.7759/cureus.4660

**Published:** 2019-05-14

**Authors:** William R Bloom, Jonathan K Ramsey, Matthew P Ohr

**Affiliations:** 1 Ophthalmology and Visual Science, The Ohio State University Wexner Medical Center, Columbus, USA

**Keywords:** intraocular foreign body, ocular siderosis, traumatic cataract, ocular trauma

## Abstract

Intraocular foreign bodies (IOFBs) can present in an insidious manner. A 20-year-old male presented with gradual visual loss in the right eye over a six-month period. He was found to have a dense cataract. During examination he was noted to have a small, healed corneal scar and subtle iris heterochromia. Further questioning revealed a previously undisclosed metal-on-metal hammering injury concerning for an IOFB. B-scan ultrasonography was inconclusive and CT studies confirmed the presence of IOFB. The patient underwent a combined cataract extraction with intraocular lens implantation with a pars plan vitrectomy, removal of IOFB, and endolaser. He had an excellent visual outcome, despite developing siderosis. A high index of suspicion should be raised for any asymmetric cataract formation, especially in younger patients. Careful examination for findings such as healed corneal scars or iris heterochromia may aid in diagnosing previously undisclosed injuries.

## Introduction

Intraocular foreign bodies (IOFBs) account for 18%-41% of all open globe injuries [1-3]. These injuries often affect younger individuals who work in industrial workplaces where metal fragments and other small materials have potential to penetrate the eye, causing damage that may threaten vision and disrupt the structure of the eye [4-5] . We report a case of ocular siderosis and traumatic cataract secondary to retained IOFB in a young male following ocular trauma in the workplace.

## Case presentation

**Figure 1:**
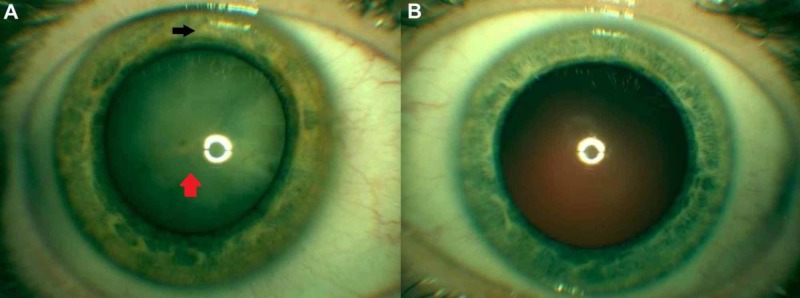
External slit lamp photography of the right eye and left eye (A-B). (A) Shows a clinically significant heterochromic iris (black arrow), a corneal scar, and a dense cataract (red arrow) suggestive of ocular siderosis secondary to intraocular foreign body in the right eye. (B) Shows no clinically significant abnormalities in the left eye.

**Figure 2:**
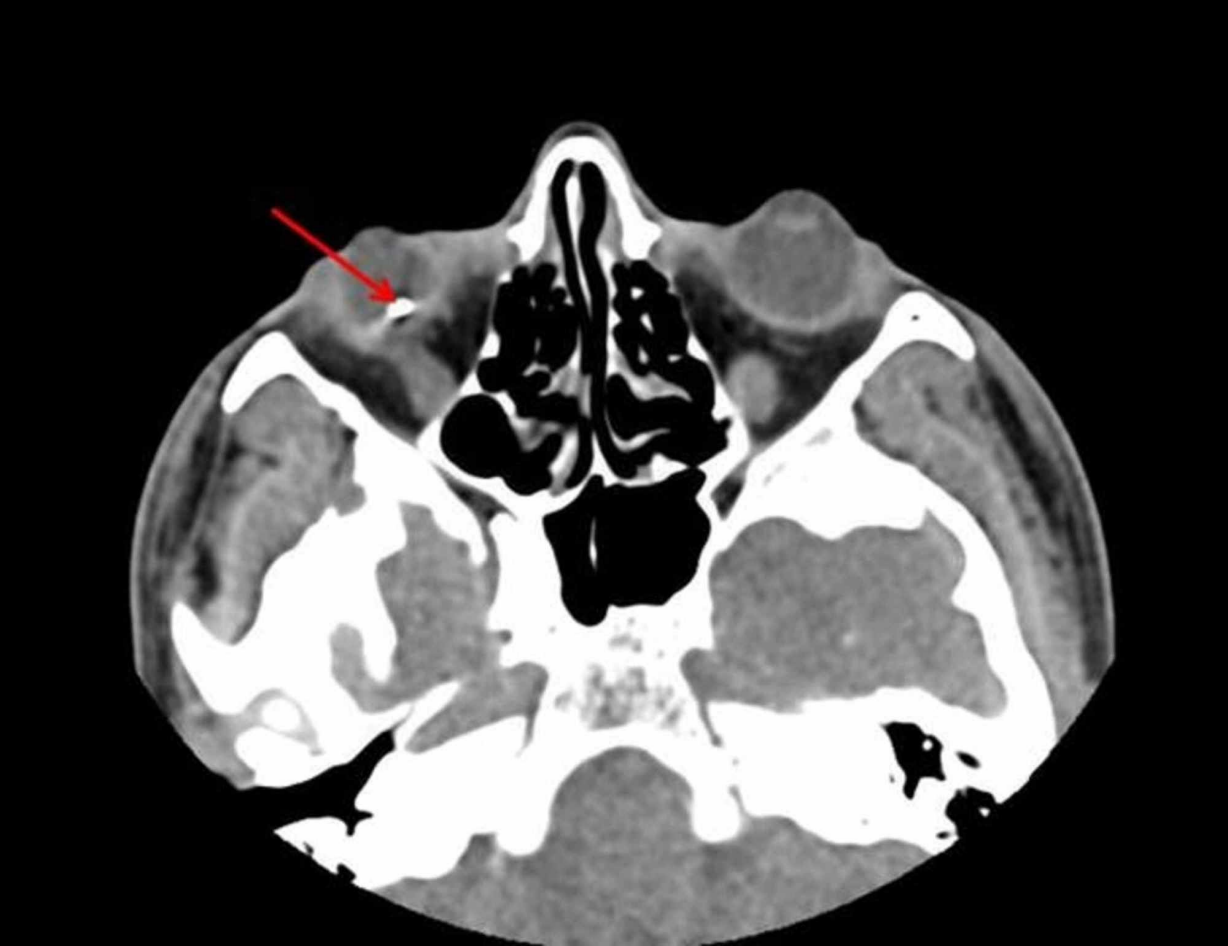
CT scan without contrast of the orbits confirming the presence of a retained intraocular foreign body in the right eye. Red arrow points to the presence of an intraocular foreign body in the right eye.

A 20-year-old male presented with decreased vision over six months and was found to have a cataract. His visual acuity (VA) at presentation was hand motion (HM) in the affected eye. Slit lamp examination of the right eye (OD) revealed a minimally reactive, heterochromic iris, a corneal scar, and a dense, mature cataract suggestive of an IOFB with secondary siderosis (Figure 1A) and no clinically significant abnormalities in the left eye (Figure 1B). Further questioning revealed a previously undisclosed history of ocular trauma that occurred while hammering metal-on-metal without eye protection. B-scan ultrasonography was inconclusive for IOFB. The patient was sent for a CT scan, which confirmed the presence of an IOFB (Figure 2). Surgery was performed, which consisted of a combined phacoemulsification with intraocular lens implant, pars plana vitrectomy, removal of IOFB, and endolaser to the impact site. 

One day after surgery, his VA without correction was 20/60 OD and there was no sign of post-operative infection. At one-week follow-up, VA improved to 20/25 OD without correction and no other ocular complications developed. At one month after surgery, VA showed continued improvement to 20/20 OD without correction.

**Figure 3:**
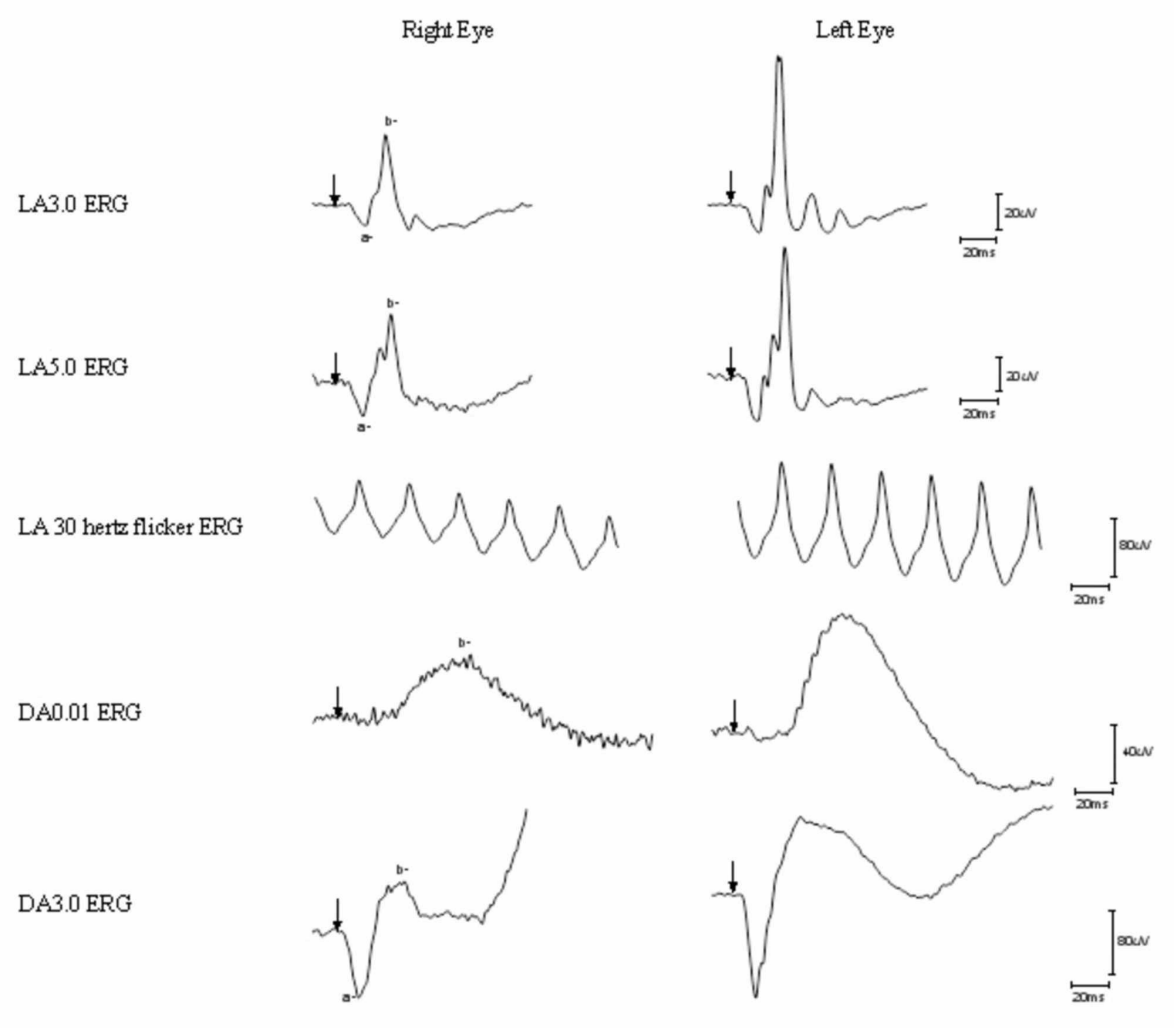
Full field electroretinogram demonstrating asymmetry of the right and left eye. Arrow, flash onset; a, a-wave; b, b-wave; LA, light adapted; DA, dark adapted; μV, microvolt; ms, millisecond. Right eye (left side) and left eye (right side) electroretinograms in a patient with ocular siderosis. Unable to obtain reliable results for the DA10.0 electroretinogram (not shown) due to excessive blinking. LA3.0/5.0 is the cone electroretinogram. LA 30 hertz flicker is the flicker electroretinogram. DA0.01 is the rod electroretinogram. DA3.0 is the rod/cone electroretinogram.

Electroretinogram (ERG) performed three months after surgery demonstrated clinically significant abnormal rod and cone functions in the right eye while the left eye revealed normal retinal function, suggestive of siderosis in the right eye (Figure 3). Post-operative spectral domain optical coherence tomography of the macula showed normal foveal contour and no clinically significant abnormalities. 

## Discussion

Historically, the time between injury and surgical intervention was thought to play a crucial role in outcome success after injury from an IOFB. However, more recent reports have found that the time between metallic IOFB injury and removal may not be as critical as previously thought [6-8]. In 1999, Jonas et al. reported that removal of IOFB within 24 h resulted in significantly less morbidity [9]. However, a more recent study, published in 2007, found no significant advantage to final VA for earlier surgical intervention to remove an IOFB [8].

Iron-containing, metallic IOFBs may lead to the development of siderosis [10]. Siderosis is caused by the deposition of ferritin particles aggregates, known as siderosomes, in the cytoplasm of ocular epithelial cells [11]. In situations where siderosis is secondary to retained IOFB, removal of the IOFB in the early stages is important to avoid visual morbidities after traumatic ocular injuries [12]. Earlier removal of IOFB may also reduce the incidence of endophthalmitis and proliferative vitreoretinopathy in patients with penetrating ocular injuries [13-15]. While siderosis may not develop in all cases of IOFB, it should be considered in patients who present with cataract, heterochromia, and/or pupillary mydriasis after ocular trauma [12, 16]. ERG can be used to quantify retinal toxicity secondary to siderosis, often displaying rod-cone functional anomalies [17] . Findings on full field ERG that suggest siderosis include decreased B-wave to A-wave ratios, progressive decreased B-wave amplitudes, and overall subnormal ERG amplitudes [12, 18]. If a diagnosis of siderosis is suspected, urgent removal of IOFB is recommended to improve the visual prognosis [19].

In one study evaluating 96 eyes that underwent surgery to remove a metallic IOFB, 40% of eyes required a second operation, with retinal detachment as the most common indication for reoperation. In the same study, a final VA of 20/50 or better was only observed in only 26% of eyes, while 50% of eyes had final VA outcomes of 20/200 or worse [6]. Development of endophthalmitis is a significant predictor of poor final VA outcomes, especially in patients with delayed IOFB removal [3].

## Conclusions

In summary, this is an example of an excellent visual outcome after surgery for a traumatic cataract and ocular siderosis treated with cataract extraction, intraocular lens implant, and IOFB removal. In many cases, patients presenting with traumatic ocular injuries resulting in a retained IOFB and siderosis do not show continued VA improvement. In this case, the patient presented with HM vision that improved to 20/20 one month after surgery. No other additional surgeries or subsequent ocular complications occurred after initial treatment for this patient. Ocular injuries among people working industrial jobs are a known risk. Many workers choose not to wear eye protection. Protective eyewear is critical to avoid ocular trauma, thus patient education and increased awareness are essential.
